# Short-Term Material Characterization by Electrohydraulic Incremental Extrusion through Micro Channels

**DOI:** 10.3390/ma14030525

**Published:** 2021-01-22

**Authors:** Lasse Langstädtler, Sebastian Schnabel, Marius Herrmann, Christian Schenck, Bernd Kuhfuss

**Affiliations:** 1 Bremen Institute for Mechanical Engineering—Bime, Badgasteiner Str. 1, 28359 Bremen, Germany; schnabel@bime.de (S.S.); herrmann@bime.de (M.H.); schenck@bime.de (C.S.); kuhfuss@bime.de (B.K.); 2 University of Bremen, 28359 Bremen, Germany; 3 MAPEX Center for Materials and Processing, University of Bremen, 28359 Bremen, Germany

**Keywords:** impulse forming, high-throughput, material testing, electrohydraulic forming, micro massive forming

## Abstract

Conventional testing procedures for characterizing the mechanical behavior of materials require intense preparation in geometry and in the handling of the samples to apply specific stress conditions. Furthermore, these procedures are time consuming. In a novel method for high-throughput development of new material, spherical and cylindrical micro samples should also be tested within a short time. For mechanical testing, the samples need to be exposed to specific types of stress. As most conventional testing procedures are not applicable, new testing procedures are demanded. The incremental electrohydraulic extrusion of micro samples through micro channels is a new testing procedure that was introduced for short-term material characterization. Loading energy is used to cause shock waves that incrementally push the samples through the forming die. The resulting deformation progress is measured between the forming steps. In this research, process simulations are used for channel design and material flow analysis. The designed channels that cause specific stress in samples are realized by stacking elements radially or axially. The stacking enables sample access for measurement and unloading and ensures good machinability of the forming channels. New testing cases for short-term characterization of cylindrical as well as spherical micro samples by electrohydraulic extrusion are presented according to monotone tensile, compression, and torsion testing. Furthermore, production-related testing and cyclic load testing are introduced by incremental electrohydraulic extrusion. By measuring the deformation due to the dependence on supplied energy, flow curve equivalents are determined that correspond to values from conventional material testing procedures.

## 1. Introduction

A novel method for the high-throughput development of evolutionary structural materials was introduced by Ellendt and Mädler [1]. Spherical and cylindrical micro samples were produced in a short time by single droplet solidification [2] and selective laser melting of various compositions [3]. After chemical composition and modification by mechanical as well as heat treatment, these micro samples were tested by simplified procedures [4]. One of these procedures is the mechanical testing by electrohydraulic incremental extrusion. Shock waves push the micro samples through a forming channel while the extrusion process is measured. From these measurements, descriptor values are derived that correlate with established material values.

Massive forming, such as extrusion, is conventionally performed with at least two rigid die parts: a stamp and die. There are only a few micro massive forming processes with only one rigid die part used for rubber pad forming [5] or electromagnetic forming [6]. However, in these processes, sheet-bulk metal forming (an overview on sheet-bulk metal forming is given in [7]) was performed by intense local material flow in sheet metal. In conventional micro massive forming, tolerances between the stamp and die are very low, which results in time- and cost-intensive tooling and the risk of abrasion [8]. Even negative clearances are used for fine blanking [9]. In micro extrusion with a small stamp diameter in deep cavities, hardened stamps tend to break from high forming forces due to the material’s limitations. In other cases, to withstand the mechanical load on material characterization in compression testing, as presented by Sonnenberg and Clausen [10], micro samples are deformed between a compression stamp with a diamond insert and an aluminum pressure plate. As rigid stamps are unable to adapt to diameter changes, sequential load cases for forming are realized in transfer systems using several stamp-and-die sets. This additionally increases handling expenses, which is a main challenge in micro forming [11].

Electrohydraulic forming is a high-speed process, which is established for macro sheet metal forming applications. The novel process of electrohydraulic incremental extrusion with small spherical [12] as well as cylindrical [13] samples with initial diameters from 0.6 mm to 2 mm was introduced. Forming was successfully performed with different sample materials like bronze and aluminum. Shock waves adapted to the changing shapes of the sample and die. The aspect ratio of the forming dies was increased. A short-term material characterization was planned at several forming stages along a forming channel. These forming channels were designed to have a high aspect ratio of channel length to channel diameter as well as channel length to sample length. As the manufacturing of channels with changing diameters is challenging, assembling them from forming channel stages was investigated [14].

The contribution is based on the work of Langstädtler [15]. To enable different short-term characterization cases and to extend the gathered material properties, new channel stages that cause different base stress/strain conditions are investigated in this work. As single stages represent monotone testing (tensile, compression, and torsion), multi stages are introduced for combined production-related as well as cyclic testing in one channel. However, the stress/strain conditions are not monotonous at all. The aim is to replicate the conditions of conventional tests and deliver significant stress components as expected in the conventional tests. For this, the process is simulated in simplified 3D models to provide a deeper insight into the material flow and the resulting strain fields. The tooling, process control, and measurement of different testing cases, as well as the transfer from the measurement data to the flow curves equivalents, is described.

## 2. Materials and Methods

**Figure 1:**
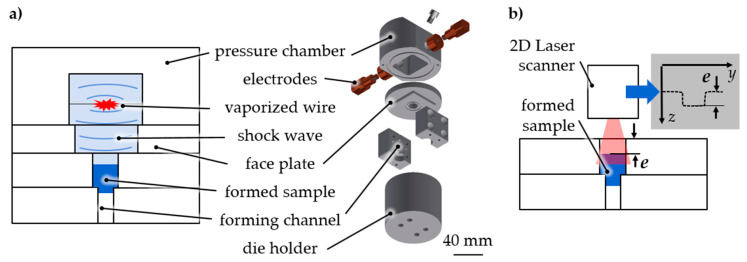
Short-term material characterization by electrohydraulic incremental extrusion. (**a**) Incremental extrusion by shock wave, (**b**) measurement of the extrusion depth *e* by laser line triangulation.

The general material characterization was performed as follows (Figure 1). A pulse generator was used with a maximum loading energy of *E*_C_ = 1800 J, a capacity of C = 100 µF, and a maximum loading voltage of *U*_0_ = 6 kV. The discharge was initiated over a single ignitron (National Electronics NL508/NL508A, Richardson Electronics, LaFox, IL, USA) resulting in peak currents of *I*_max_ = 5–10 kA. The electrical discharge of the current pulse generator provided the energy for vaporization. An aluminum (Al99.5) wire with a length of *l*_w_ = 20 mm and a diameter of *d*_w_ = 0.3 mm was placed between two copper within a water-filled pressure chamber. The discharge led to the vaporization of the wire and plasma. Shock waves arose that caused a pressure increase—up to several GPa (gigapascals) were possible—within a few microseconds. The shock wave power is influenced by the loading energy of the pulse generator and the material as well as by the geometrical properties of the vaporized wire [12]. The shock wave acted as an adaptable stamp on the micro samples (Figure 1a). Depending on the applied load, the friction conditions between the sample and die, and the sample material’s behavior, the sample was pulse pushed into the forming element of the die. The progress was measured with a laser scanner as extrusion depth *e* (Figure 1b) or a comparable geometric value due to the testing case.

**Figure 2:**
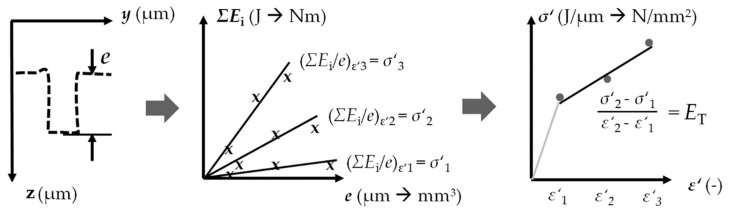
Material characterization example—tensile test equivalent [15].

Consecutive vaporized wires incrementally transmitted energy to the micro sample in several small extrusion steps. The extrusion depth *e,* which is a measure of the forming result, is a function of the cumulated energy *ΣE*_i,_ which is a measure of the cumulated forming effort. The supplied loading energy was transferred to forming energy. The plastic strain in the sample depends on the geometry of the forming element in the channel. The relative energy *ΣE*_i_/*e* was determined as the stress equivalent *σ*′ for different stress and related to different strain *ε*′. The resulting flow curves equivalent was considered equivalent to the flow curve (Figure 2). As a first approximation of the flow curve equivalent *σ*′(*ε*′), the tangent modulus *E*_T_ can be considered part of the bilinear hardening.

**Figure 3:**
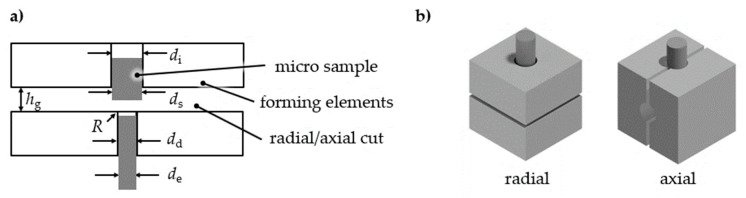
Extrusion die (**a**) basic configuration, (**b**) die assembling for change in forming direction [15].

To address different stress/strain conditions in a single testing stage, different forming elements were designed (Figure 3a) and mounted in a die holder placed under the pressure chamber. The parameters of the initial die diameter *d*_i_ and extrusion diameter *d*_d_ as well as the gap distance *h*_g_ are the basic parameters that yield tensile strain and stress. To withstand the high forming forces for forming steel, the extrusion channel was modified with hardened extrusion inserts (drill bushes). A change in forming direction (redirection) results in shear strain (Figure 3b). The base forming elements were made of steel S355.

Testing was performed on cylindrical and spherical samples with an initial diameter of *d*_s_ = 0.8–2.0 mm. With an energy increment of *E*_i_ = 200–800 J for each incremental extrusion step in the vaporization of aluminum wires, the final deformation was reached with *ΣE*_i_—depending on the test equivalent with several or at least one extrusion step. The edge radius *R* varied in different test set-ups. The configurations of different test equivalents under different stresses are found in Table 1.

**Figure 4:**
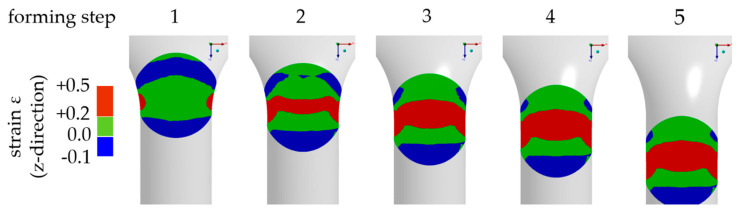
Simulation example of extrusion spherical micro samples (AlSi12; *d*_s_ = 800 µm; *d*_e_ = 700 µm; R = 1.00 mm; α = 40°).

Forming stages were designed to cause different stress/strain conditions in micro samples. Forming simulations in Ansys 3D (Workbench 17) with bilinear hardening (material: AlSi12; *E* = 75 GPa; *R*_P0.2_ = 70 MPa; *R*_m_ = 150 MPa [16]) were used to determine suitable forming channel base geometries. Samples were simulated as a deformable mesh into around 18,000 tetrahedron elements (Ansys element type solid 187) to ensure sufficient accuracy. The forming channel was assumed to be a rigid body. Hardening was set isotropically and was strain rate independent. Contact conditions between the sample and die were defined by the Coulomb friction model with a friction coefficient of *µ* = 0.1. The process effect of the electrohydraulic shock waves was implemented as the initial velocity of the sample before die contact. This simplification reduced the computing time and reproduced the adaptive force effect of the shock waves. The impact velocity was set to *v*_i_ = 600 m/s resulting in kinetic energy depending on sample mass. As an example, simulations show the generation of regions in the sample that have significant positive total strain in the forming direction of the tensile test equivalent, which verifies the suitability of the geometry (Figure 4).

## 3. Results

### 3.1. Tensile Test Equivalent

**Figure 5:**
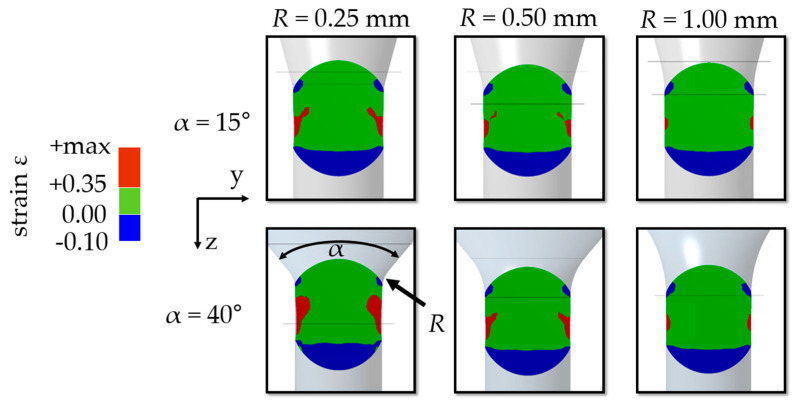
Simulation for variation of channel geometry (AlSi12; *d*_s_ = 800 µm, *d*_e_ = 700 µm; forming step 4).

As confirmed by the simulations, a diameter reduction caused tensile strain during incremental extrusion (Figure 5). The channel was designed to maximize positive strain fields. An increase of strain was reached with a higher opening angle (*α*) and a lower radius (*R*). Smooth more monotone fields were realized by a decrease of angle and an increase of radius.

**Figure 6:**
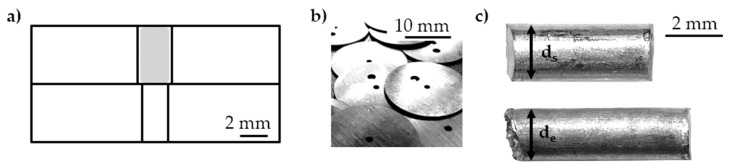
Tensile test equivalent set-up (**a**) scheme, (**b**) forming elements, (**c**) initial (**top**) and completely extruded (**bottom**) sample [11].

The diameter reduction was realized by stacking forming elements of different diameters (Figure 6). The adaptable stamp followed the change of die diameter. The initial diameter of the cylindrical Al99.5 samples was *d*_s_ = 2.00 mm. The completely extruded samples had a diameter of *d*_e_ = 1.80 mm. Although the axially assembled dies were not preloaded, no flow of material into the gap between the forming elements could be observed.

**Figure 7:**
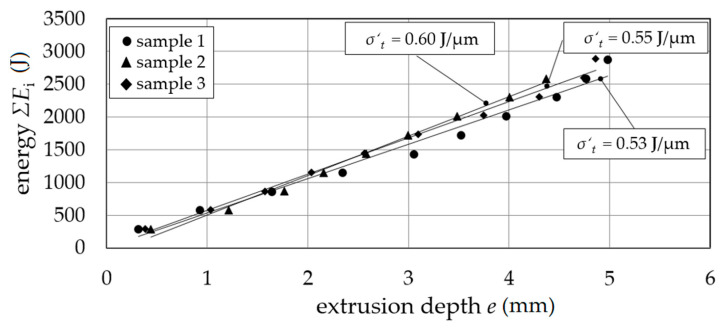
Tensile stress equivalent *σ*′_t_; extrusion depth *e* as a function of energy *ΣE*_i_.

In the single stage diameter reduction, the energy *ΣE*_i_ as a function of the extrusion depth *e* showed an average tensile stress equivalent to *σ*′_t_ = 0.56 J/µm for different samples 1 to 3 (Figure 7) for constant tensile true strain equivalent *ε*′_t_ calculated with extruded (*d*_e_) and initial sample diameter (*d*_s_) *ε*′_t_ = |2 ln(*d*_e_/*d*_s_)| ≈ 0.22.

**Figure 8:**
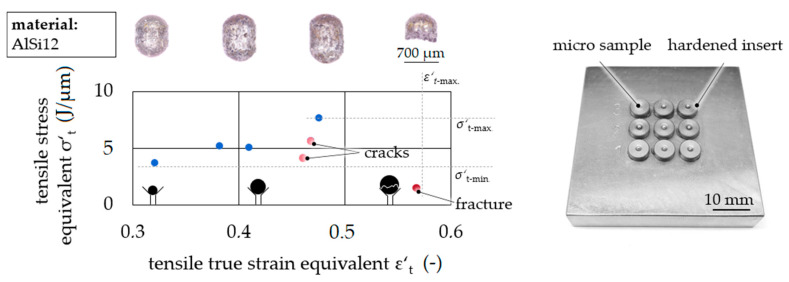
Flow curve equivalents—tensile stress equivalent σ′_t_ as a function of tensile true strain equivalent *ε*′_t_.

The parallel characterization of spherical AlSi12 micro samples was introduced by a multiforming die holder (Figure 8). With one shock wave, nine samples were tested with different true tensile strain equivalent values (*ε*′_t_) due to different initial sample diameters. The flow curve equivalent is given by the tensile stress equivalent σ′_t_ as a function of the true tensile strain equivalent *ε*′_t_. Evaluating the method, the ratio of characteristic values *σ*′_t-min._/*σ*′_t-max._ ≈ 0.5 agreed with the yield strength ratio *R*_e_/*R*_m_ ≈ 0.5 due to the yield strength *R*_e_ and tensile strength *R*_m_ from conventional tensile testing for AlSi12 [16]. Furthermore, a qualitative validation of simulation and experiment was given by the congruence of the resulting samples’ geometry as well as the failure behavior. The elongation at break was slightly higher than for quasi-static testing, which was expected to be caused by the high strain rate.

### 3.2. Compression Test Equivalent

**Figure 9:**
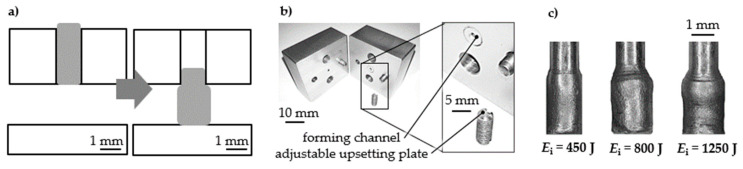
Compression test equivalent set-up (**a**) scheme, (**b**) radial openable forming channel, (**c**) deformed sample each in one forming step [15].

The material characterization by compression of micro samples was performed (Figure 9). Cylindrical Al99.5 samples had an initial diameter of *d*_s_ = 1.00 mm. The initial channel diameter was *d*_i_ = 1.00 mm, and the gap distance was *h*_g_ = 2.00 mm. Short-term compression was performed in one step with different energies *E_i_* = 450 J, 800 J, and 1250 J. Micro samples were accelerated over the gap distance by adapting the shock wave and performing a partial free compression. The micro samples deformation increased with the supplied energy. The resulting deformation and decrease in cylindricity increased with energy *E*_i_ = 1250 J. The given free-forming behavior of the samples agrees with the observations by Sterionow [17] about the dynamic effects in free compression behavior of samples under increased forming speed due to the impact on the compression plate and high strain rates. Opening the die after forming and unloading the sample without sticking was possible.

(1) $${\bar{d}}_{e}=\frac{1}{5}\overset{5}{\underset{i=1}{\sum }}d_{ei}$$

**Figure 10:**
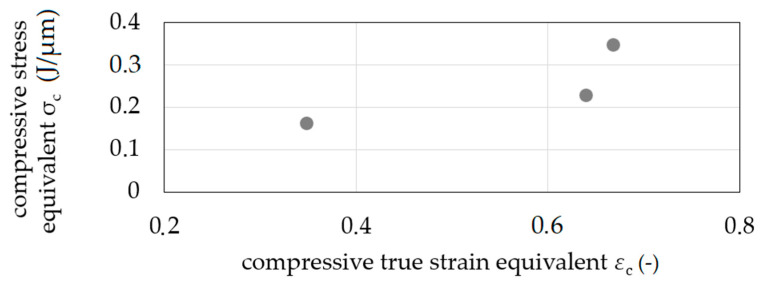
Flow curve equivalents—compression stress equivalent σ′_c_ as a function of compressive true strain equivalent *ε*′_c_.

The increase of sample diameter was measured at five positions, and the mean value was calculated with Equation (1). The compressive stress equivalent σ′_c_ was calculated for the performed compression with different compressive true strain equivalents *ε*′_c_ calculated by *ε*′_c_ = 2 ln(${\bar{d}}_{e}$/*d*_s_) (Figure 10). The resulting curve is considered to be a compression flow curve equivalent.

### 3.3. Shear Test Equivalent

**Figure 11:**
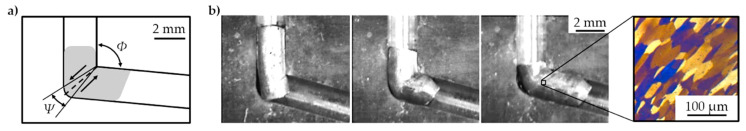
Shear test equivalent set-up (*Φ* = 105°) (**a**) scheme, (**b**) sample (Al99.5) deformation (from left to right after extrusion step one, four, and seven) within the axial openable forming channel and grain structure in the opening plane [15].

A short-term characterization by shear testing (torsion) was performed with four dies with different shear angles *Φ* of 90° to 135° based on the novel method equal channel angular blasting (ECAB), where shear stresses are induced—as established by equal channel angular extrusion (ECAE) [18] or equal channel angular pressing (ECAP) [19], respectively—by electrohydraulic extrusion (Figure 11). The shearing of cylindrical aluminum samples (Al99.5) with *d*_s_ = 2 mm and length *l*_s_ = 5 mm through the channel with a constant channel diameter *d*_e_ = 2 mm and *d*_d_ = 2 mm by ECAB was possible in multiple extrusion steps (Figure 11b). Shear strain *γ* is calculated by [20] for ECAP, as given by Equation (2) with transition radius *Ψ* and shear angle Φ in degrees.

(2) $$\gamma =2\cot\left(\frac{\Psi }{2}+\frac{\Phi }{2}\right)+\Psi \cdot cosec\left(\frac{\Psi }{2}+\frac{\Phi }{2}\right)$$

The values of transition radius *Ψ* were geometrically determined with *Ψ*(90°) = 36.9°, *Ψ*(105°) = 33.0°, *Ψ*(120°) = 27.8°, and *Ψ*(135°) = 21.6° in a CAD 3D model of the testing set-up.

**Figure 12:**
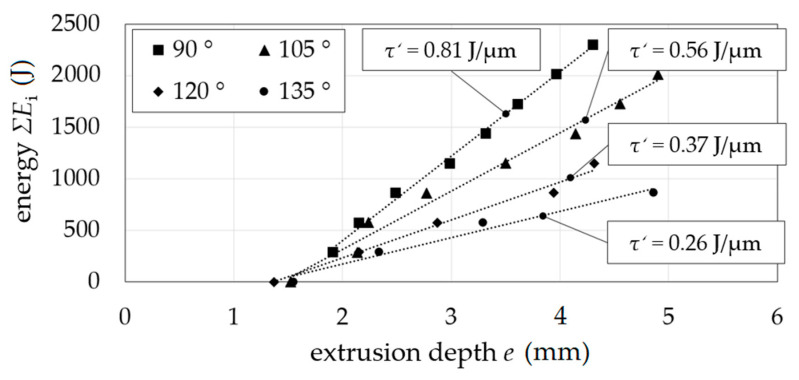
Shear stress equivalent τ′; extrusion depth *e* as a function of energy *ΣE*_i_ for shearing angles *Φ*.

The shearing of grain was verified by the grinding pattern (Figure 11b). The opening between the extrusion steps was enabled. No material flow into the gap between the die sides with a radial gap was detected. Incremental energy *ΣE*_i_ as a function of extrusion depth *e* showed a constant slope Σ*E*_i_*/e* depending on the shear angle *Φ* as can be seen in Figure 12. A decrease of shear angle resulted in an increase of slope, which corresponds to the change in the pressing force of conventional equal channel angular pressing at different shear angles.

**Figure 13:**
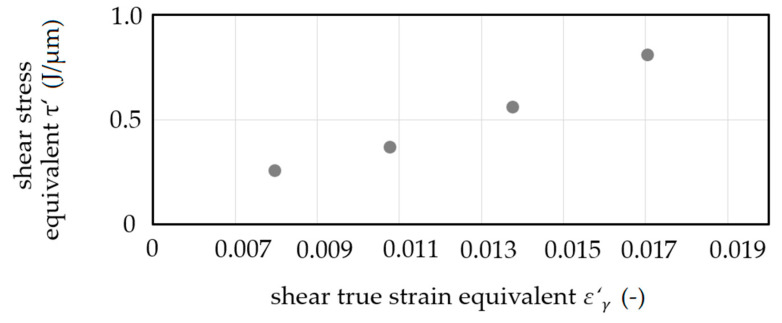
Flow curve equivalents—shear stress equivalent τ′ as a function of shear true strain equivalent *ε*′_*γ*_.

The shear true strain equivalent *ε*′_*γ*_ is introduced as *ε*′_*γ*_ = ln(*γ* + 1). Shear stress equivalent *τ*′ increased with the increase of shear true strain equivalent *ε’*_*γ*_ (Figure 13). This increase corresponds to the idea of a flow curve equivalent in the sense of load dependent strain hardening. An increase of the resulting strain hardening with an increase in shear true strain equivalent was confirmed by quasi-static compression tests at an Hegewald and Peschke inspect table 100 testing machine (compression of *ε* = 1.0). With a maximum force of *F*_c_ ≈ 1.75 kN for compression in the radial direction on the unformed sample, the maximum force for compression of formed samples increased by 15% for samples sheared by *ε*′_*γ*_ = 0.008 up to 23% for samples sheared by *ε*′_*γ*_ = 0.017.

### 3.4. Real Part Approximation

**Figure 14:**
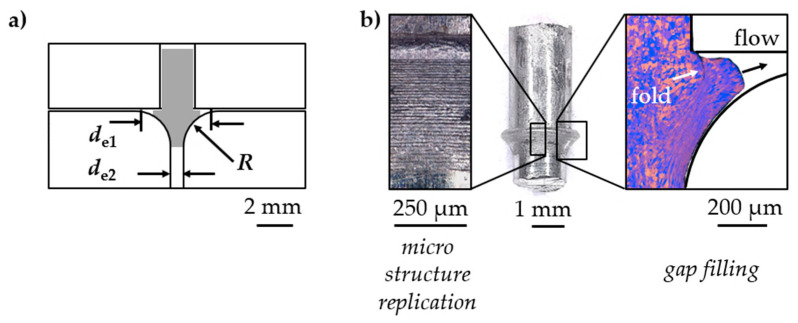
Exemplary real part approximation (**a**) scheme, (**b**) deformed sample (**middle**)—surface micro structure replication (**left**)—grain formation and form-filling (**right**).

For material characterization related to the production of real parts, the forming channel was designed to emulate the multi-axial strain of a production process. Cylindrical aluminum samples (Al99.5) were tested by applying one, single incremental extrusion step with an incremental energy of *E*_i_ = 800 J. The initial channel diameter was *d*_i_ = 2.00 mm, sequentially increased to a maximum of *d*_e1_ = 3.70 mm while being reduced afterwards to an extrusion channel diameter of *d*_e2_ = 1.70 mm. Hence, the material was stressed by compression with a partial backflow resulting in shearing. A subsequence tensile stress was applied (Figure 14). By intensive forming in a diameter increase, high strain was reached in the sense of material-forming limit testing. Furthermore, in the sense of production-related material testing, form-filling properties by micro structure replication and gap filling were addressed (Figure 14b). As a result, the test introduces the potential of incremental electrohydraulic extrusion in micro channels for recreating and simulating the production of parts. The tested material withstood intense strain. Due to material flow and friction conditions, 70% gap filling was performed.

### 3.5. Cyclic Stress Test Equivalent

**Figure 15:**
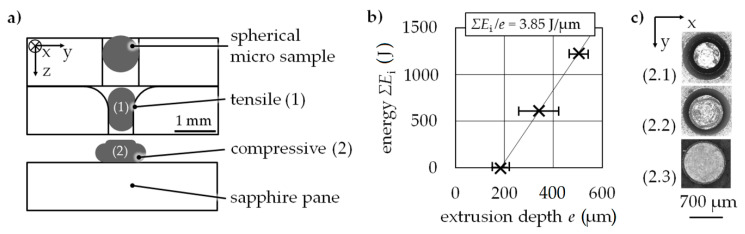
Cyclic test equivalent (**a**) scheme, (**b**) energy *ΣE*_i_ as a function of extrusion depth *e* for the tensile stage testing 100Cr6, (**c**) microscope images (bottom view) of the compression stage in sequenced forming steps each *E*_i_ = 800 J.

A cyclic load test equivalent was realized by sequenced tensile (1) and compressive (2) forming stages (Figure 15). The whole sample is first drawn and afterwards pressed. A further means of cyclic testing is the continuous increase of strain. A radial channel cut is used for the upsetting stage with a gap of *h*_g_ = 1.00 mm. A sapphire pane was used here as a compression plate that gives optical access for measurement for later testing scenarios. Spherical 100Cr6 micro samples in an annealed state with an initial sample diameter of *d*_s_ = 0.79 mm were successfully extruded through a micro channel with a diameter of *d*_e_ = 0.70 mm for tensile stress. The energy increased as a function of extrusion depth (Figure 15b).

**Figure 16:**
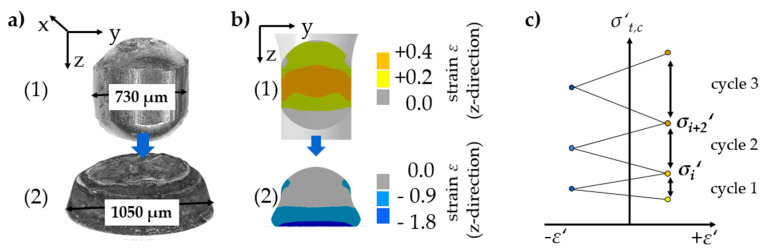
Cyclic test equivalent (**a**) experiment of single cycle, (**b**) simulation of single cycle, (**c**) example of cyclic test equivalent curve.

In the second stage, compressive stress was applied successfully on a micro sample by incrementally forming it on a sapphire pane. The forming of the 100Cr6 micro sample on the sapphire pane is shown in Figure 15c. For each incremental energy (*E*_i_ = 800 J) an increase in the sample’s diameter resulted in a contact zone between the sample and compression plate. Using simulations in Ansys, the resulting stresses and strains and the size of the strain fields can be conducted (Figure 16).

For material characterization, several cycles are sequenced in a forming channel. With each cycle between positive (tensile) and negative (compressive) strain, stress equivalence increases. Furthermore, the change Δ*σ*′ = *σ*_2_′ − *σ*_1_′ increases by kinematic hardening. The given values in this scheme correspond to the maximum values in the hysteresis loops of conventional cyclic testing. These values are, for example, usable for Chaboche material modelling.

## 4. Conclusions

In this paper, incremental electrohydraulic extrusion through micro channels was introduced as material characterization method for high-throughput material development of evolutionary structural materials. Material characterization was divided into three base cases (tensile, compression, shearing) and combined for production-related as well as cyclic testing. Test scenarios were conducted according to established testing procedures as the tensile test equivalent, the compression test equivalent, and the shear test equivalent as well as the production-related test equivalent and the cyclic stress test equivalent under the use of a simulation model. The simulations agreed with the experimental investigations and enabled the design of different testing cases. The following conclusions can be deduced:

- different test equivalents were realized in the forming channel by single stages;
- single stages are combinable with low distance to realize multiaxial/production-related testing;
- single stages are combinable with increased distance to realize multi-stage cyclic testing;
- flow curve equivalents were determined for different test equivalents; and
- a cyclic stress test equivalent was validated by experiments and simulation.

The method was finally proven by exemplarily comparing material value equivalents with material values from conventional testing methods.

## 5. Outlook

With the aim of conducting multiple tests along the forming channels, besides the questions of how sensitive the characterization is to changes in the material and how this affects the determined flow curves, future work deals with the question of which material values can be deduced and how interactions between the stages influence these values. Following constraints are expected to influence multi-stage testing:

- the stress–strain stage sequence;
- the distance between stages; and
- the samples’ dimensions, material, and surface.

**Figure 17:**
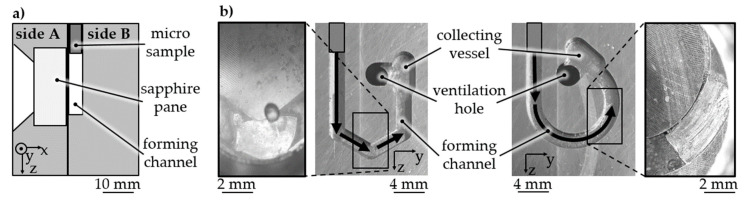
Improvement of forming channels and characterization method (**a**) winding channels with local and continuous change of direction, (**b**) 3 × 120° ECAP and curved channel—side view scheme of forming die with measurement through optical access.

Due to the demand for measurability as well for channels with increased length, future work deals with the investigation of base geometries like winding channels (Figure 17a). Forming tests proved the suitability for forming into multi-stage channels with an aspect ratio >10 and a change in the forming direction. Material samples were analyzed by optical measurement through a sapphire pane, which is one side of a forming channel (Figure 17b). The measurement technique conducted through an inspection window during electrohydraulic incremental forming is well described by Stöbener et al. in [21]. The determined flow curves will be correlated to further material characterization results. Furthermore, forming channels are planned to be used for the measurement of friction conditions in forming processes.

## Figures and Tables

**Table 1:** Configuration for different test equivalents.

Stress Equivalents:	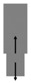 Tensile *σ*′_t_	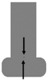 Compression *σ*′_c_	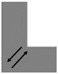 Shear *τ*′	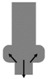 Real Part Approx.	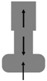 Cyclic
initial sample diameter *d*_s_ (mm)	2.00, 0.80	1.50	2.00	2.00	0.79
initial die diameter *d*_i_ (mm)	2.00	1.75	2.00	*d*_d1_ = 2.00 *d*_d2_ = 3.60	1.00
extruded sample diameter *d*_e_ (mm)	1.80, 0.70, 0.60	0.00	2.00	1.70	0.70
extrusion diameter *d*_d_ (mm)	1.85, 0.70, 0.60	0.00	2.00	1.70	0.70
gap distance *h*_g_ (mm)	0.00	1.00	0.00	0.00	1.00
cut	axial	axial	radial	axial	axial
radius *R* (mm)	0.00; 1.00	0.00	0.00	1.00	1.00
die material	S355	S355; hard. insert	S355	S355; hard. insert	S355; hard. insert
formed material	Al99.5, AlSi12	Al99.5	Al99.5	Al99.5	100Cr6

## Data Availability

The data presented in this study are available on request from the corresponding author.
